# AUTOMATED MEASUREMENT OF MUSCLE DENSITY ON COMPUTED TOMOGRAPHY (CT) PREDICTS ALL-CAUSE MORTALITY IN OLDER ADULTS

**DOI:** 10.1093/geroni/igz038.3234

**Published:** 2019-11-08

**Authors:** Leon Lenchik, Ryan Barnard, Robert D Boutin, Stephen B Kritchevsky, Ashley A Weaver, Fang-Chi Hsu

**Affiliations:** 1 Wake Forest School of Medicine, Winston-Salem, North Carolina, United States; 2 UC Davis School of Medicine, Sacramento, California, United States

## Abstract

The purpose was to examine the association of paraspinous muscle density (CT surrogate of myosteatosis) with all-cause mortality in 6803 men and 4558 women, age 60-69 years (mean age 63.6) in the National Lung Screening Trial. Our fully-automated machine learning algorithm: 1) selected the appropriate CT series, 2) chose a single CT image at the level of T12 vertebra, 3) segmented the left paraspinous muscle, and 4) recorded the muscle density in Hounsfield Units (HU). Association between baseline muscle density and all-cause mortality was determined using Cox proportional hazards models, adjusted for age, race, body mass index, pack years of smoking, and presence of diabetes, lung disease, cardiovascular disease, and cancer at enrollment. After a mean 6.44 ± 1.06 years of follow-up, 635 (9.33%) men and 265 (5.81%) women died. In men, lower muscle density on baseline CT examinations was associated with increased all-cause mortality (HR per SD = 0.90; CI = 0.83, 0.99; p=0.03). Each standard deviation (7.8 HU) decrease in muscle density was associated with a 10% increase in mortality. In women, the association did not reach significance.

